# Scaling Down for Efficiency: Medium-Sized Transformer Models for Protein Sequence Transfer Learning

**DOI:** 10.1101/2024.11.22.624936

**Published:** 2024-11-24

**Authors:** Luiz C. Vieira, Morgan L. Handojo, Claus O. Wilke

**Affiliations:** a Department of Integrative Biology, The University of Texas at Austin, Austin, TX, United States of America

**Keywords:** ESM2, pLM embeddings, Feature compression, Transfer learning

## Abstract

Protein language models such as the transformer-based Evolutionary Scale Modeling 2 (ESM2) can offer deep insights into evolutionary and structural properties of proteins. While larger models, such as ESM2 15B, promise to capture more complex patterns in sequence space, they also present practical challenges due to their high dimensionality and high computational cost. We systematically evaluated the performance of all ESM2 models across many biological datasets to determine the impact of model size on transfer learning. Surprisingly, larger models do not always outperform smaller ones, especially when data is limited. Medium sized models, such as ESM2 650M, exhibited consistent performance, falling only slightly behind the 15B parameter model despite being over 20 times smaller. Additionally, we compared various methods of embedding compression to identify the most effective approach, and we found that mean embeddings consistently outperformed other compression methods. Our results show that ESM2 650M with mean embeddings offers an optimal balance between performance and efficiency, making it a practical and scalable choice for transfer learning in a variety of biological applications.

## Significance Statement

This work challenges the common belief that larger language models always yield better results, here in the context of protein biochemistry. By systematically comparing transformer models of different sizes in transfer learning tasks, we demonstrate that medium size models, such as ESM2 650M, frequently perform as well as larger variants, specially when data is limited. These findings provide a more efficient strategy for machine learning-based protein analysis and promote the broader accessibility of AI in biology. Smaller, more efficient models can help democratize advanced machine-learning tools, making them more accessible to researchers with limited computational resources.

Machine learning (ML) has significantly advanced the field of protein biochemistry (1–4). By leveraging large datasets and sophisticated models, ML techniques have improved the accuracy of protein property predictions (5–7). Transformer models, such as Evolutionary Scale Modeling 2 (ESM2) (8), a pre-trained masked protein language model (pLM), have been particularly impactful due to their ability to capture valuable evolutionary relationships of protein sequences. The pLM models learn about protein properties during pre-training, where the models are trained to predict residues that have been masked in their input sequences (9, 10). The pre-training process causes the models to encode knowledge about protein biochemistry and protein evolution in the models’ internal representations, known as embeddings, which encapsulate the biochemical characteristics of individual amino acids as well as complex higher-order interactions that reflect both local and global structural and functional properties of proteins (11, 12). The capacity of pLMs to learn rich representations of proteins from vast amounts of unlabeled sequence data and subsequently apply this knowledge to secondary supervised tasks, a process known as transfer learning (13), has enabled their application in various downstream tasks including functional annotation, mutational effect analysis, and the design of novel proteins and peptides (14–17). Finally, these embeddings can also be used for homology search, in particular when sequences are short or highly diverged (18, 19).

There has been a trend toward increasing the size of pLMs, following similar advancements in natural language processing (NLP), where model scaling laws predict that model performance systematically increases with increasing model size and commensurate increase in pre-training data (20, 21). Models such as the largest ESM2 variant, with 15 billion parameters, and more recently ESM3, with a staggering 98 billion parameters, have demonstrated that scaling model size can enhance performance by capturing more complex relationships in protein sequences (7, 12). Although models such ESM3 can deliver notable gains in accuracy, their high computational cost hinders their development and utilization. For example, fine-tuning these large models is highly computationally demanding (22), limiting their use primarily to private industry or highly resourced laboratories. Recently, some groups have begun questioning whether larger models are the best option or if alternative methods such as improving data quality, increasing data quantity, or extending the number of training steps, could boost smaller models’ performance (23). This shift in thinking could provide a more accessible and cost-effective alternative for the broader research community, as smaller models might offer sufficient performance for many scientific applications at a lower cost.

Here, we systematically evaluate the impact of model size on transfer learning, considering models with parameter counts ranging from 8 million to 15 billion across various biological datasets. These datasets include 12 different metrics calculated from proteins in the PISCES dataset (24) and 41 deep mutational scanning (DMS) datasets (25). Our findings suggest that the effectiveness of transfer learning is influenced by the choice of embedding compression method, dataset size, and model size. Larger models perform better with larger datasets, underscoring the need for sufficient data to maximize their utility. However, when data is limited, medium-sized models perform comparably to, and in some cases outperform, larger models. Furthermore, while the impact of compression methods varies depending on dataset type, mean embeddings generally yield excellent results across tasks. These findings emphasize the importance of selecting model size and embedding method based on the unique characteristics of the data and task to enhance both the efficiency and accuracy of transfer learning in protein applications.

## Results

### Mean embeddings outperform all other compression methods.

Despite the general success of transformer model embeddings, their high dimensionality poses challenges in practical applications, particularly in transfer learning scenarios (26, 27). In most scenarios, embeddings need to be compressed before any downstream prediction tasks are performed. The most commonly used compression strategy is simply averaging embeddings over all sites (mean pooling), though other methods such as max pooling, beginning-of-sequence (BOS) tokens, inverse Discrete Cosine Transform (iDCT), or Principal Component Analysis, have also been proposed (18). Because mean pooling averages over the contributions of all sites in a sequence, this strategy may not retain all critical, in particular in deep mutational scanning (DMS) applications, where one or a few amino acid changes can significantly affect a protein’s thermodynamic stability or enzymatic activity (28).

**Fig. 1.**
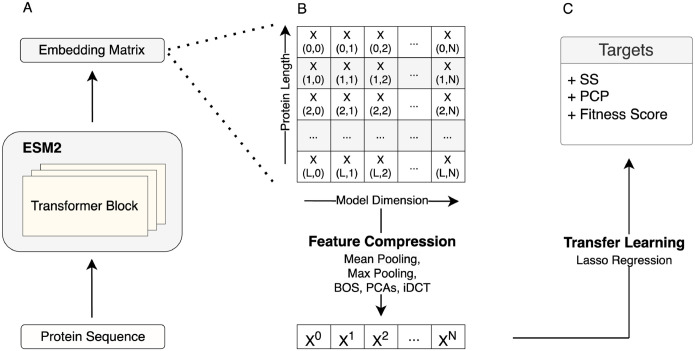
Schematic view of the transfer learning approach used throughout this work. A) Protein representation (embeddings matrix) extraction using the ESM2 model. B) Embeddings matrix compression using various methods including mean pooling, max pooling, BOS, PCAs, and iDCT. C) Transfer learning to predict downstream tasks, such as secondary structure(SS), fitness, and physical-chemical properties (PCPs)

To assess the effect of compression method on transfer learning performance, we systematically explored a wide range of different compression methods. We benchmarked the compression methods on 40 DMS datasets (which tend to be datasets of single or few point mutations relative to a reference sequence) and on a set of diverse protein sequences taken from PISCES (A Protein Sequence Culling Server), for which we calculated a variety of target variables including physicochemical properties (PCPs), instability index, amino acid frequencies, and secondary structure frequencies. In our prediction pipeline, for a given protein sequence, we extracted embeddings from the last hidden layer of the ESM2 150M model, compressed them via one of the methods, and then used the compressed embeddings as input features in regularized regression models (LassoCV) to predict the target of interest (Figure 1).

For both the DMS datasets and the PISCES sequences, we generally found that mean pooling outperformed all other compression methods we considered (SI Appendix, Fig. S1 and Fig. S2). However, the extent to which mean pooling performed better depended on the type of data. For diverse protein sequences, mean pooling was strictly superior in all cases, and in many cases by a wide margin (Fig. S2). By contrast, for DMS data, some alternative compression methods, including max pooling, iDCT, and PCA, were slightly better than mean pooling on some datasets, even if on average mean pooling was superior (Fig. S1). These findings suggest that mean pooling does to some extent average out effects from individual sites, yet on the whole it still tends to perform better than the alternatives.

**Fig. 2.**
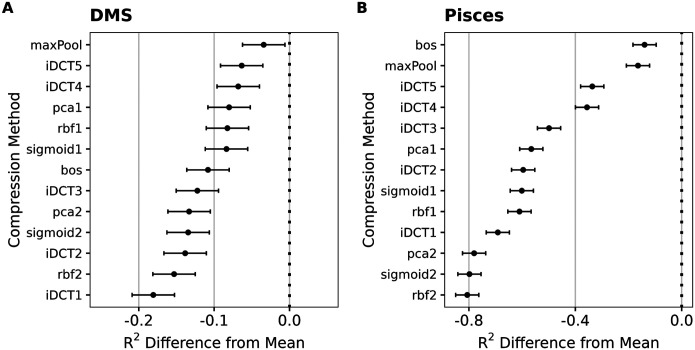
Mean reduction in *R*^2^ when embeddings are compressed with methods other than mean pooling. A) Results for DMS data. B) Results for diverse protein sequences (PISCES data). In all cases, the y-axis represents different compression methods and the x-axis shows the resulting difference in *R*^2^. Dots represent the fixed effects estimates from mixed-effects modeling, and error bars represent 95% confidence intervals.

To summarize these findings more systematically, we fit linear mixed-effects models to the results of all compression methods and either all DMS datasets or all PISCES prediction targets, respectively. In this setup, we treated the type of compression as a fixed effect and the dataset/prediction target as a random effect, to isolate the impact of the compression method while accounting for the variability between datasets. This analysis showed that mean pooling was, on average, significantly better than all other alternatives we considered, in both types of datasets (Figure 2). For DMS data, mean pooling led to an increase in variance explained (measured by *R*^2^ from the regularized regression, calculated on a hold-out test set) between 5 and 20 percentage points (Figure 2A). For diverse protein sequences, the difference was even more stark, where mean pooling led to an increase in variance explained between 20 and 80 percentage points (Figure 2B).

In aggregate, these results show that mean pooling is strictly superior in transfer-learning applications where the input sequences are widely diverged, and it performs well also with DMS data. Therefore, for our subsequent assessment of model size on transfer learning, we only considered mean pooling throughout.

### Moderately sized models perform well in transfer learning.

We next turned to the effect of model size. We considered all 6 ESM2 models, ranging from 8 million parameters (EMS2 8M) to 15 billion parameters (ESM2 15B). We also included the older model ESM1v with 650 million parameters, which was developed specifically for variant effect prediction. Importantly, because ESM1v only accepts sequences up to 1,022 residues, we excluded any DMS datasets with longer proteins from the main analysis, which reduced our DMS data to 35 distinct datasets. Results on the remaining datasets but excluding EMS1v are presented in SI Appendix, Fig. S3.

**Fig. 3.**
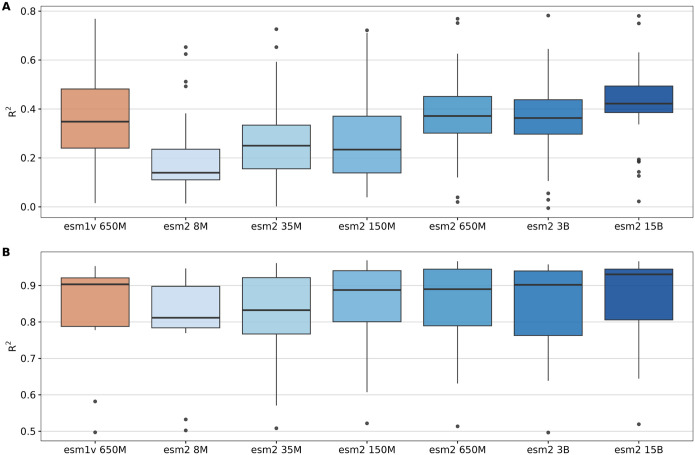
Impact of ESM Model size on transfer learning. A) LassoCV regression results using ESM mean embeddings for 35 DMS datasets. B) LassoCV regression results for 12 targets from the PISCES dataset. The x-axis represents different ESM model sizes: ESM1v 650M (orange) and ESM2 8M, 35M, 150M, 650M, 3B, and 15B (blues). The y-axis displays *R*^2^ for each task.

We found that overall, larger models tended to yield better results for transfer learning tasks across both datasets, as expected (Figure 3). However, improvements for very large models (3 or 15 billion parameters) were moderate or small. The medium-size model ESM2 650M consistently performed well across many targets—especially in DMS analyses, where it slightly outperformed ESM2 3B despite being six times smaller (Figure 3A). Additionally, for some DMS datasets, ESM2 650M even surpassed the performance of the largest model ESM2 15B (SI Appendix, Fig. S4). On the PISCES dataset, all models with 150M parameters or more delivered comparably strong performance (Figure 3B and SI Appendix, Fig. S5). These findings indicate that, for the datasets evaluated, the ESM2 650M model strikes an ideal balance between computational efficiency and predictive power, offering robust results in the context of transfer learning.

Although ESM1v was specifically designed and trained on UniRef90 for variant effect prediction, its limitation to sequences no longer than 1,022 residues restricts its applicability. To evaluate sequences exceeding this length, we turned to ESM2 models. Even though these models were also pretrained with a maximum sequence length of 1,022, due to ESM2’s rotary attention mechanism it can in principle handle longer sequences. However, this capability remains underexplored. To enable embeddings for these larger proteins, we adjusted the “max sequence truncation” parameter in the ESM2 extraction script. We found this part of our analysis inconclusive, as none of the larger sequences achieved high predictability scores (SI Appendix, Fig. S3A). However, when we tested six additional proteins with lengths within the training range, performance was not consistently better (SI Appendix, Fig. S3B), suggesting that sequence length alone may not drive predictive accuracy. At a minimum, we can state that there is no strong evidence that EMS2 cannot be used with sequences longer than 1,022 residues.

### Sample size drives transfer learning performance in ESM models.

**Fig. 4.**
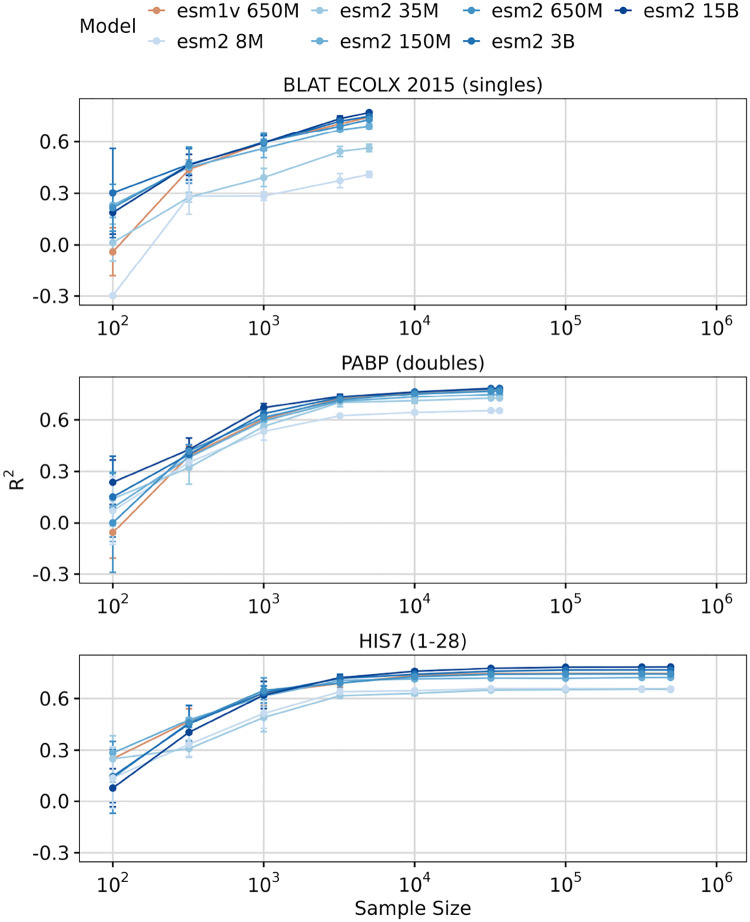
Effect of sample size on transfer learning. Results of LassoCV regression on three DMS datasets, using downsampled subsets ranging from 100 to the maximum number of samples in the dataset. The y-axis represents *R*^2^ scores, and the x-axis the sample sizes tested. The colored lines represent different ESM model sizes: ESM1v 650M (Orange) and ESM2 8M, 35M, 150M, 650M, 3B, and 15B (Blues).

Next, we investigated whether sample size impacts transfer learning performance. To explore this question, we selected three DMS datasets with good model performance (*R*^2^ > 0.6 for the larger models), large size (> 1000 observations), and differing complexity (single, double, and multiple mutations). We then progressively downsampled each dataset into subsets, ranging from 100 observations to the full dataset size. For each subset, we ran LassoCV regression with five-fold cross-validation. We found that smaller datasets negatively affected transfer learning performance, resulting in reduced accuracy for all three datasets (Figure 4). Notably, aside from the smallest two ESM2 models (8M and 35M parameters), all models performed comparably for sample sizes below 10^4^. This result suggests that if sample size is limited, medium size models can perform as well as larger models. However, when sufficient data is available (≥ 10^4^ observations), the 15B model can outperform small and medium-sized models (Figure 4).

We hypothesized that meaningful protein features are distributed across more dimensions in larger models, leading to improved performance as the number of retained features increases. To test this hypothesis, we plotted the number of features retained by LassoCV for the three DMS datasets. We found that the ESM2 15B model starts outperforming the smaller models when it starts utilizing a greater number of features (SI Appendix, Figure S6). Interestingly, the HIS7 dataset was the only one that showed a saturation in the number of features being used, plateauing around 1,000 features for sample sizes in excess of 10^4^. By contrast, the number of retained features in the other datasets continued to increase until the maximum dataset size was reached, suggesting that with more samples, performance could improve further. These results suggest that the larger embedding spaces provided by larger models cannot be fully taken advantage of by moderately sized datasets of fewer than 10^4^ observations, and this may be one of the main reason moderately sized models are frequently sufficient and perform just as well as the largest models.

### Transfer learning is limited by data quality.

**Fig. 5.**
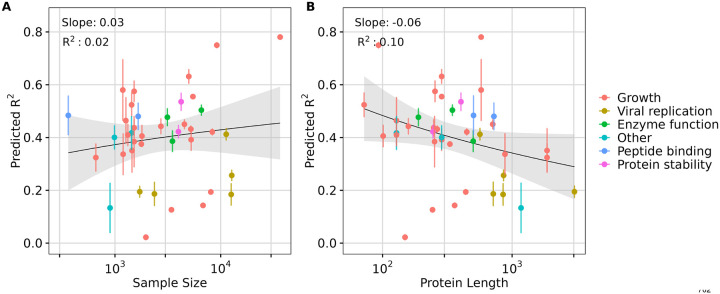
Effect of sample size and protein length in transfer learning. A) Effect of **sample size** on transfer learning using ESM2 15B model embeddings quantified by the regression results (slope and *R*^2^ score). B) Effect of **protein length** on transfer learning using ESM2 15B model embeddings quantified by the regression results (slope and *R*^2^ score). The color of the dots represents different data measurement type.

Since the pre-ceding analysis was performed by downsampling, where we expected prediction performance to systematically decline for more extreme ranges of downsampling, we next asked whether a similar relationship between dataset size and prediction accuracy could be observed across all DMS datasets. In addition, we also considered protein length and the type of data measured. We fit linear regression models with DMS dataset size and protein length as independent variables and model performance (*R*^2^ score) as the dependent variable, evaluating all ESM2 models. Our analysis revealed that the proportion of variability explained by sample size was minimal (for example, *R*^2^ = 0.02 for ESM2 15B, Figure 5A), indicating that while performance may improve with larger datasets, sample size is not the primary driver of transfer learning effectiveness in the 40 DMS datasets analyzed. Notably, most datasets had a sample size of over a thousand (SI Appendix, Fig. S7), a number close to optimal for transfer learning, as suggested by our downsampling results (Figure 4). By contrast, protein length accounted for a greater proportion of the variability (for example, *R*^2^ = 0.10 for ESM2 15B, Figure 5B), suggesting a modest influence on model performance. Specifically, longer protein sequences, particularly those exceeding the training limit of 1,022 residues, were associated with decreased model performance in transfer learning (Figure 5A and SI Appendix, Fig. S3A). This trend was broadly consistent across model sizes (SI Appendix, Fig. S9 and S10).

We also broke down the datasets by dataset type (growth, viral replication, peptide binding, etc.) but did not see a strong trend (Figure 5). One exception were viral proteins, which tended to be among the longest sequences in the DMS data and also exhibited some of the lowest *R*^2^ scores. This observation suggests that either ESM embeddings may struggle to capture effective representations of viral proteins specifically, or that these embeddings generally lack representational power for larger proteins. We note that the vast majority of DMS datasets (all but four) used sequencing as the primary measurement method, and frequency measurements via sequencing are subject to numerous sources of noise, including sampling errors, PCR amplification errors, and genetic drift during competition (29). These factors may partially explain the limited predictive power we observed for several of the datasets. When datasets are inherently noisy no machine learning model can possibly make good predictions on them.

## Discussion

We have evaluated the performance of ESM2 embeddings across various model sizes (from 8 million to 15 billion parameters) in transfer learning tasks on a wide range of different biological datasets. We have found that larger models do not consistently outperform smaller ones, especially when data is limited. In fact, medium-size models, such as ESM2 150M or ESM2 650M, have demonstrated consistently good performance. This observation suggests that model size should be carefully aligned with dataset size and data type to optimize transfer learning performance. Additionally, we have evaluated different methods for compressing per-token embedding matrices, and we have found that, on average, mean embeddings consistently outperformed other commonly used compression methods. However, for fairly homogeneous DMS datasets, alternative compression methods including max pooling and iDCT did sometimes achieve better performance than mean embeddings.

Scaling large language models (LLMs) has become a trend since Kaplan et al. (30) showed that there is a power-law relationship between model size and its performance, which has driven the development of increasingly larger models (20, 21). Since the introduction of Generative Pre-Trained Transformers (GPT) (31) in 2018, LLMs have scaled to trillions of parameters (32). This tendency has also extended to biology, with the latest protein language models (pLMs) reaching an astonishing 98 billion parameters (7). Although larger pLMs have demonstrated enhanced performance by capturing more complex relationships in protein sequences (12), a recent study has shown that as models scale, they become more prone to overfitting, often preferring to predict the wild-type residue in masked tasks (23). This observation aligns with the known LLM scaling laws, which suggest that performance improvements from scaling are only achieved if the amount of training data is scaled as well (30). As ESM2 models were not trained with data scaled across different model sizes, their performance gains may have been limited, in particular for the larger models.

In addition to concerns about overfitting, the use of larger models is also hindered by their extensive computational demands. Moreover, the costs do not end with pre-training, as companies often spend more energy during inference (33), increasing the environmental footprint for years to come. Given the burden of pre-training LLMs, fine-tuning has emerged as a promising alternative. However, even fine-tuning remains highly computationally intensive (22). As a result, it is reasonable to question whether larger models are the optimal solution, or if alternative strategies, such as improving data quality, increasing data quantity, or extending the number of training steps could enhance the performance of smaller models (23).

Fine-tuning pLMs has enhanced predictions across various tasks (34). This approach, however, necessitates a sufficient amount of training data (35, 36). In cases where data is limited, transfer learning becomes an effective alternative (37). Traditional models such as Lasso regression prioritize simplicity (fewer features) while fitting the training data well (low error). Consequently, they often select fewer features to prevent overfitting and enhance generalization (38). However, this approach limits the ability to fully leverage the rich embeddings from the ESM2 15B model, as Lasso tends to select fewer features, especially with smaller datasets. We reason that this inefficiency arises because critical features necessary for effective transfer learning are distributed across the model’s many dimensions. Consequently, the performance of transfer learning with larger models’ embeddings was constrained when tested on smaller datasets. However, when evaluated on larger datasets containing up to half a million sequences, the 15B model outperformed all the other models, if only by a small margin.

Our study challenges the general preference for larger models in biology. In particular, it suggests that in common transfer learning applications, available datasets are too small for the largest available pLMs. While larger models can achieve good performance in principle, smaller models frequently perform similarly or deliver stable performance with fewer samples, making them more accessible and practical in the more common scenarios when dataset size is limited. Additionally, mean embeddings consistently perform well across datasets, suggesting that alternative compression methods will rarely be needed. In summary, intermediate model sizes such as ESM2 150M or 650M and mean embeddings are likely sufficient for transfer learning on the vast majority of data sets encountered in biological research.

## Materials and Methods

### A. Code availability.

All the code used to generate the metadata, embeddings, compressed embeddings and models are available at https://github.com/ziul-bio/SWAT.

### Data collection.

We used two types of datasets in our analysis. First, we obtained 41 deep mutational scanning (DMS) datasets previously curated in Ref. (25). Table 2 in their data availability section cites each original study from which we obtained the wild-type sequences to reconstruct the mutated sequences. Based on the collected data, we created FASTA and CSV files containing the mutated sequences for each protein, along with the corresponding target values of the mutations. Second, we compiled a dataset of diverse protein sequences with known structures. We downloaded the PISCES dataset (24), which contains a collection of proteins with known structures and at most 50% pairwise sequence similarity. We then filtered this dataset to include only proteins with lengths between 66 and 1022 residues (1022 is the maximum length supported by ESM2 models). From this dataset, we generated 12 target variables, including physicochemical properties such as hydrophobicity, instability index, amino acid frequencies, and secondary structure frequencies, using the peptides 0.3.2 package available on PyPI.

### Calculating protein embeddings.

We calculated protein embeddings using both the ESM2 family of protein language models (12) and the older ESM1v model (39). For all model variants, the model’s internal representation is a matrix of embeddings in **R**^*n×d*^ dimensions, where *n* represents the protein sequence length and *d* represents the embedding dimension, which differs for different model variants and generally increases for models with more parameters. For each protein sequence in our datasets, we fed the sequence into the respective model and obtained the corresponding embeddings from the final hidden layer. These embeddings were then used as input features for subsequent analyses.

For ESM2, we performed this calculation using the extract script available at the ESM2 GitHub repository https://github.com/facebookresearch/esm/blob/main/scripts/extract.py. This script allows us to define three output representations: mean representation (the embeddings averaged across the sequence length), BOS representation (the CLS token or beginning of the sequence), and per token representation (the full embedding matrix). We used this script to obtain all three representations.

Next, to efficiently compress the per token representation matrix, we employed several techniques. Initially, the features were scaled using a Min-Max Scaler to ensure consistency, with scaling applied across the sequence length. For feature compression, we utilized Principal Component Analysis (PCA) by transposing the last hidden layer representation matrix so that the sequence length became the features and the model dimensions became the samples. After applying PCA, we reversed this transformation to obtain a single vector of transformed embeddings. Similarly, we applied Kernel PCA with Radial Basis Function (RBF) and Sigmoid kernels for embeddings transformation.

Lastly, we employed an inverse Discrete Cosine Transform (iDCT) quantization method, as a method of embeddings compression, as described in the Protein Ortholog Search Tool (PROST), which has shown promise as a predictor for protein homology (18). iDCT is particularly adept at capturing fine-grained details in data while reducing dimensionality by retaining just the high frequency features enhancing the signal-to-noise ratio in the embeddings (40, 41). We applied the Discrete Cosine Transform (DCT) to the embeddings, selected the top components, and then performed the inverse DCT. This process was followed by 2D DCT compression, which further reduced the dimensionality and reshaped the array into a 1D vector. We explored various final dimensions, ranging from the original size used in PROST to larger dimensions beyond the ESM2 15B model, specifically: 220, 640, 1280, 5120, and 6400. These configurations are referred to as iDCT1, iDCT2, iDCT3, iDCT4, and iDCT5, respectively.

### Predicting Protein Fitness and Properties.

To evaluate the predictive performance of compressed protein embeddings, we used LassoCV regression due to its effective feature selection capabilities. We began by applying various compression methods to the embeddings and scaling them between −1 and 1. This scaling is crucial for both PCA and LassoCV, as unscaled features can adversely affect performance. LassoCV (38) was chosen for its ability to perform feature selection by shrinking the coefficients of less important features to zero, thereby highlighting the most relevant features for prediction. Using 5-fold cross-validation, we applied LassoCV to these scaled features and calculated performance metrics including *R*^2^, MAE, RMSE, and Spearman’s *ρ* for both training and testing sets. This approach ensures that the features selected by LassoCV contribute significantly to the model’s predictive accuracy.

### Statistical Analysis.

To assess the statistical significance of differences among compression methods, we employed linear mixed-effects models using the lme4 package in R (42). This method is well-suited for analyzing data with hierarchical or nested structures. In our analysis, the fixed effect was the compression method (e.g., mean representation, BOS token, PCA, kernel PCA, iDCT), while the random effect accounted for variability across the different datasets. To evaluate the significance of the fixed effects estimates we used the package multcomp (43) in R to calculate 95% confidence intervals around each estimate, taking into account that we were testing multiple hypotheses at once (one for each compression method).

## Supplementary Material

Supplement 1
