# Supplementary material for: Scaling down for efficiency: Medium-sized protein language models perform well at transfer learning on realistic datasets
Source: bioRxiv. 2025 Jan 28:2024.11.22.624936. Originally published 2024 Nov 24. Preprint. [Version 2] doi: 10.1101/2024.11.22.624936 (PMC11601519; doi:10.1101/2024.11.22.624936)
Supplement: Supplementary file 1 [file media-1.pdf]

Supporting Information for

Scaling down for efficiency: Medium-sized protein language models perform well at transfer learning on realistic datasets

Luiz C. Vieira, Morgan L. Handojo, and Claus O. Wilke

This PDF file includes:

Figs. S1 to S8
Table S1

Supporting Information

Table S1. Target description for PISCES dataset.

Class	Target	Definition
Amino-acid Statistics	Alanine	The fraction of alanine residues present in the protein sequence.
Amino-acid Statistics	Cysteine	The fraction of cysteine residues present in the protein sequence.
Amino-acid Statistics	Leucine	The fraction of leucine residues present in the protein sequence.
Physico-chemical Properties (PCP)	Length	Number of residues in the protein.
Physico-chemical Properties (PCP)	Charge	The theoretical net charge of a protein sequence, based on the Henderson-Hasselbach equation.
Physico-chemical Properties (PCP)	Hydrophobicity	The averaged hydrophobicity values of each residue.
Physico-chemical Properties (PCP)	Instability index	The stability of a protein based on its dipeptide composition.
Physico-chemical Properties (PCP)	Molecular Weight (Kilodaltons)	The sum of the masses of each amino acid.
Physico-chemical Properties (PCP)	Isoelectric point	The isoelectric point (pI), which is the pH at which a particular molecule or surface carries no net electrical charge.
Secondary Structure Frequency (SS)	Extended strand (beta strand)	The fraction residues in extended strand conformation.
Secondary Structure Frequency (SS)	Alpha helix	The fraction of residues in alpha helix conformation.
Secondary Structure Frequency (SS)	Coil	The fraction of residues in coil conformation.

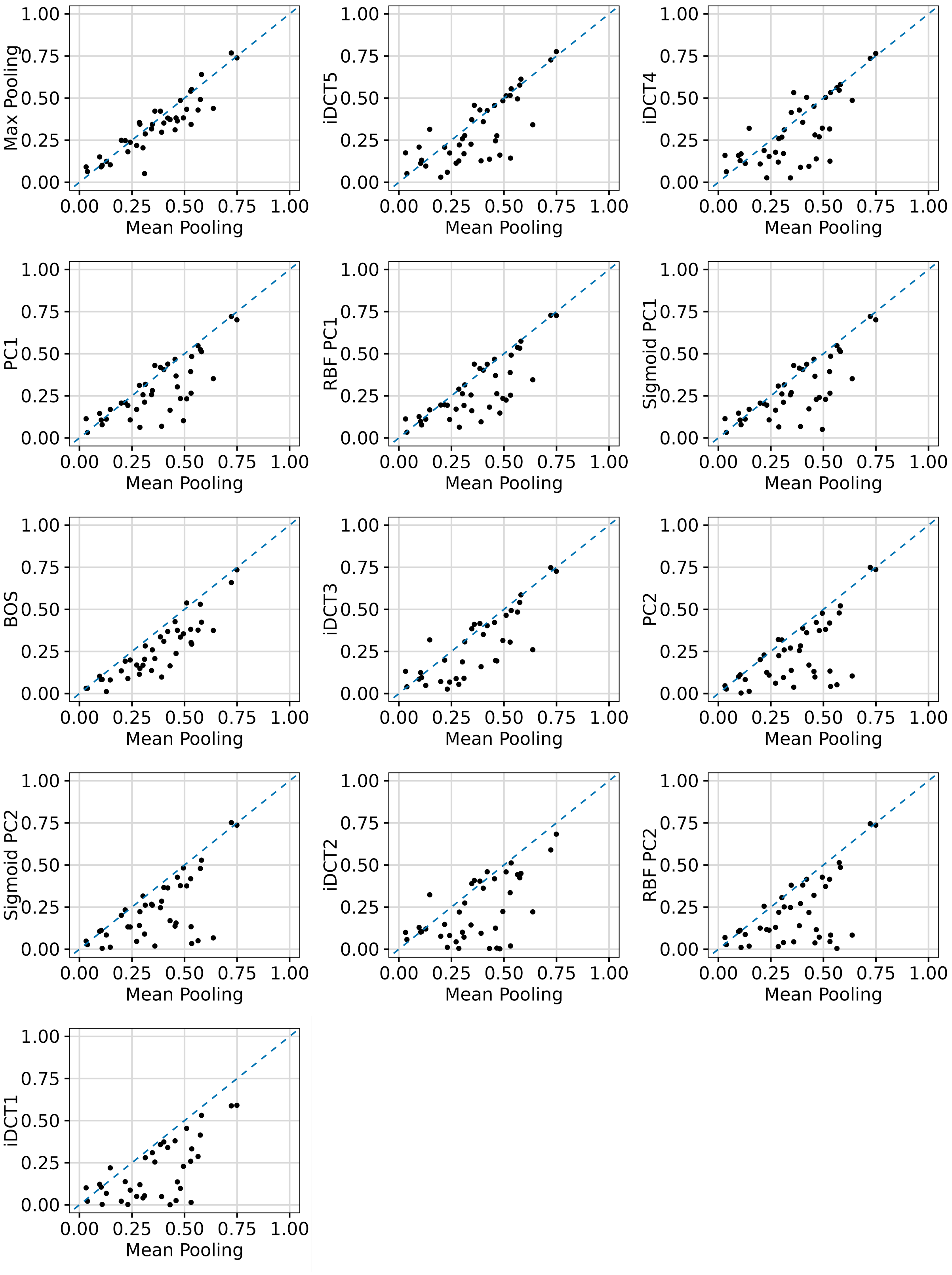

Fig. S1. Comparison of mean embeddings with 13 alternative compression methods on DMS datasets. In each plot, the x-axis represents the averaged R^2 scores from 5-fold cross-validation for the mean embeddings, while the y-axis shows the averaged R^2 scores from 5-fold cross-validation for one of the other 13 compression methods. Each dot corresponds to one of the 40 DMS datasets analyzed.

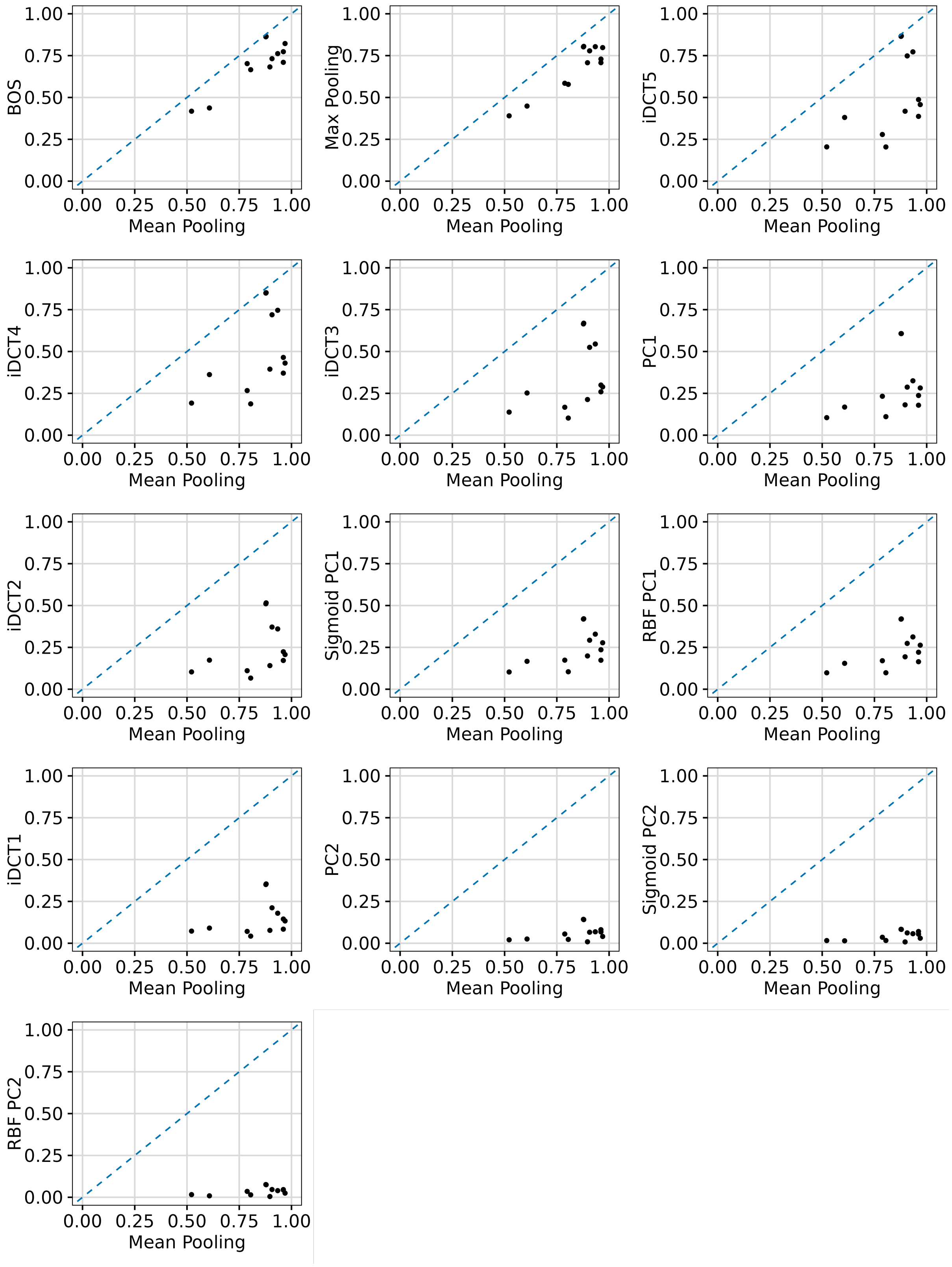

Fig. S2. Comparison of mean embeddings with 13 alternative compression methods on PISCES dataset. In each plot, the x-axis represents the averaged R^2 scores from 5-fold cross-validation for the mean embeddings, while the y-axis shows the averaged R^2 scores from 5-fold cross-validation for one of the other 13 compression methods. Each dot corresponds to one of the 12 targets analyzed from PISCES dataset (PCPs and Secondary Structure).

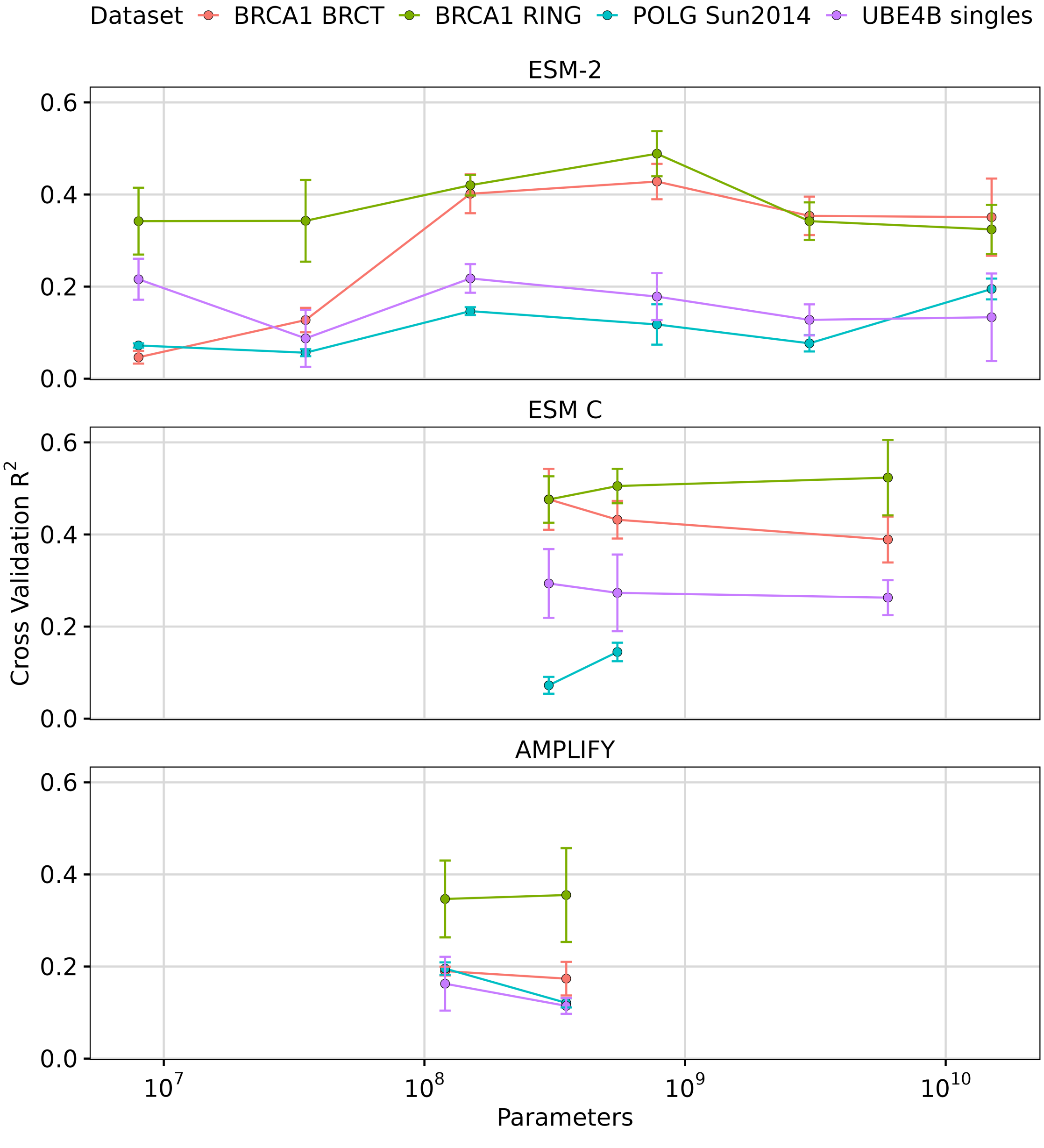

Fig. S3. Performance of pLMs on larger protein sequences. Prediction scores across 4 different DMS datasets with sequence lengths greater than 1022. The y-axis represents the averaged 5-fold cross-validation R^2 scores, and error bars represent the standard deviation. The x-axis shows the different model sizes. Colors represent individual protein datasets, varying in their lengths: 3033 (POLG Sun2014), 1863 (BRCA1 BRCT), 1863 (BRCA1 RING), 1173 (UBE4B singles).

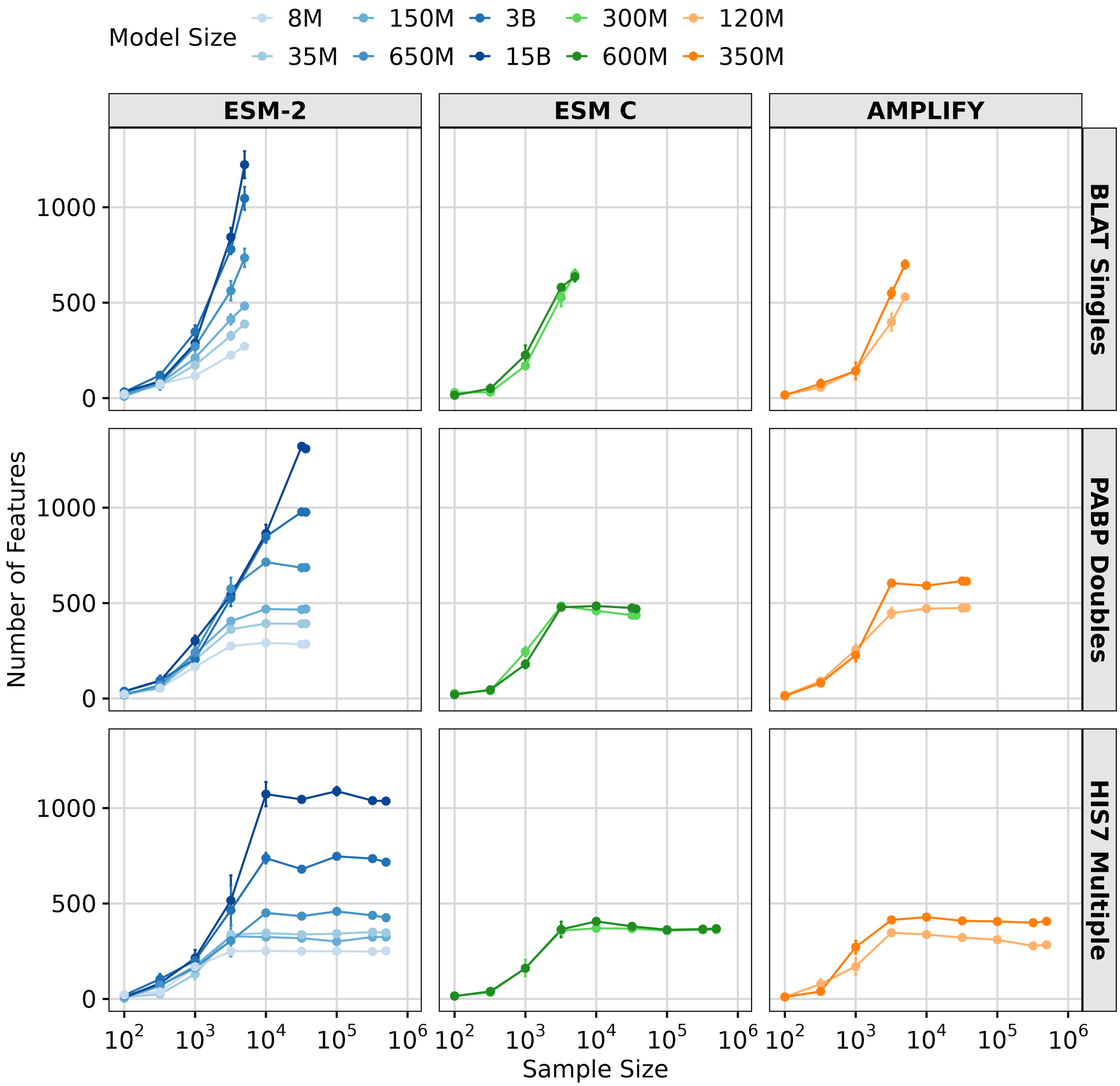

Fig. S4. Number of features boosts performance in protein prediction tasks. Number of important features identified by LassoCV regression for three DMS datasets with varying mutation complexities: single mutations (BLAT ECOLX - 2015), double mutations (PABP), and multiple mutations (HIS7 YEAST). The y-axis represents the averaged 5-fold cross-validation number of important features (or non-zero coefficients set by Lasso l1 penalization) scores, and error bars represent the standard deviation. ESM models sizes are represented by colored lines: ESM1v 650M (Orange) and ESM-2 8M, 35M, 150M, 650M, 3B, and 15B (Blues)

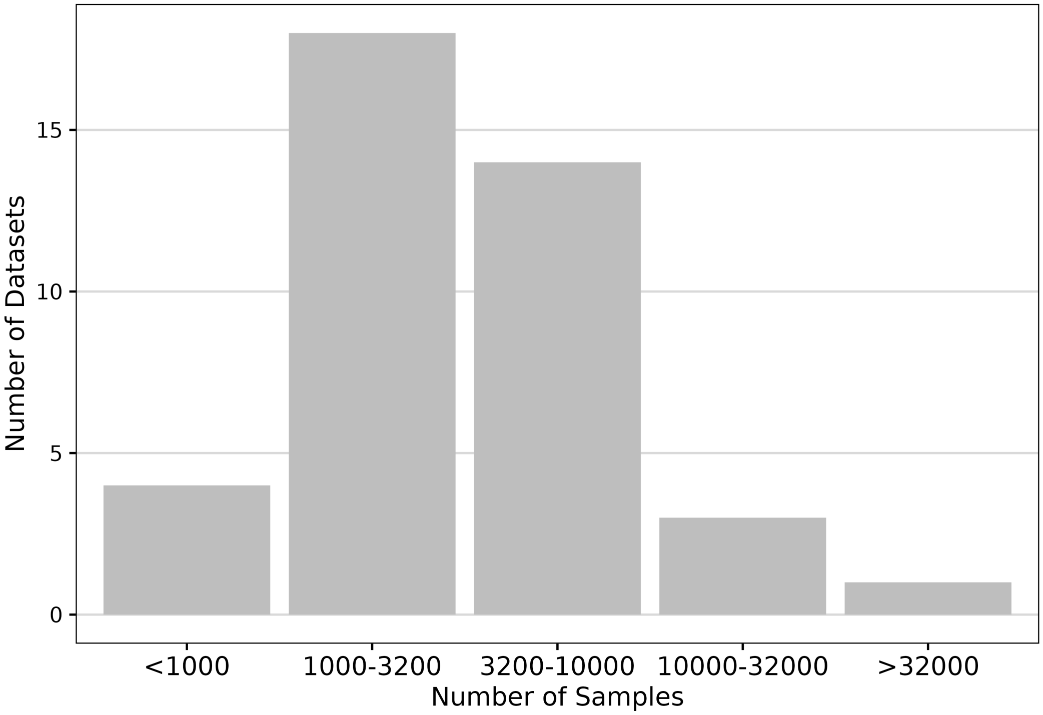

Fig. S5. Sample size distribution in deep mutational scanning (DMS) datasets.

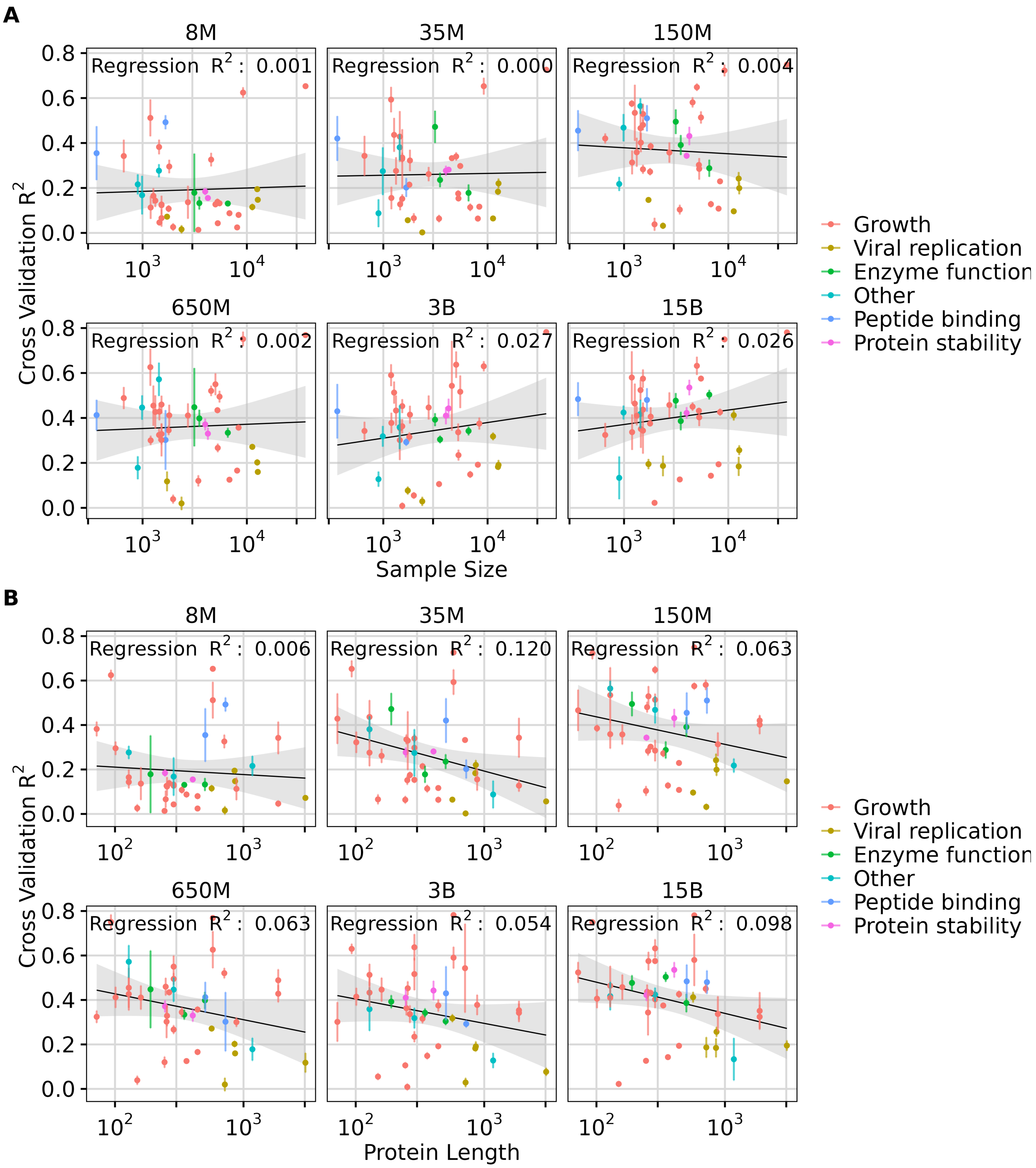

Fig. S6. Effect of sample size and protein length in transfer learning using ESM-2 embeddings. A) Effect of **sample size** on transfer learning using ESM-2 model embeddings quantified by the regression results (R^2 score). B) Effect of **protein length** on transfer learning using ESM-2 model embeddings quantified by the regression results (R^2 score). Each dot represents a dataset, and its color indicates the corresponding data measurement type. In each subplot, the y-axis represents the averaged 5-fold cross-validation R^2 scores, and error bars represent the standard deviation.

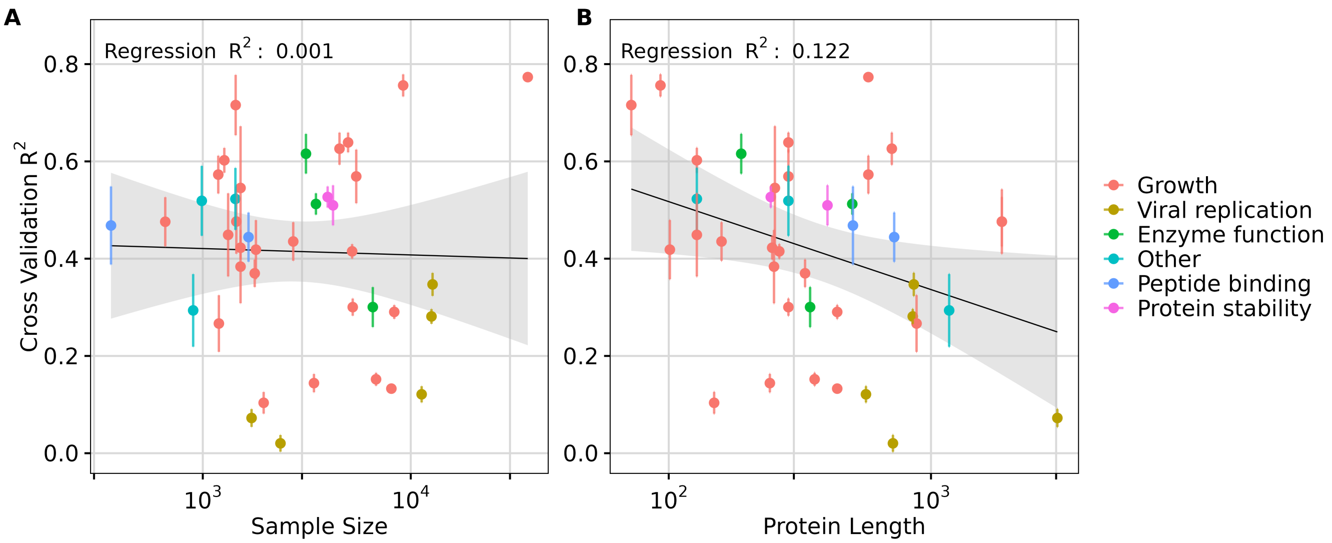

Fig. S7. Effect of sample size and protein length in transfer learning using ESM C 300M embeddings. A) Effect of **sample size** on transfer learning using ESM C 300M model embeddings quantified by the regression results (R^2 score). B) Effect of **protein length** on transfer learning using ESM C 300M model embeddings quantified by the regression results (R^2 score). Each dot represents a dataset, and its color indicates the corresponding data measurement type. In each subplot, the y-axis represents the averaged 5-fold cross-validation R^2 scores, and error bars represent the standard deviation.

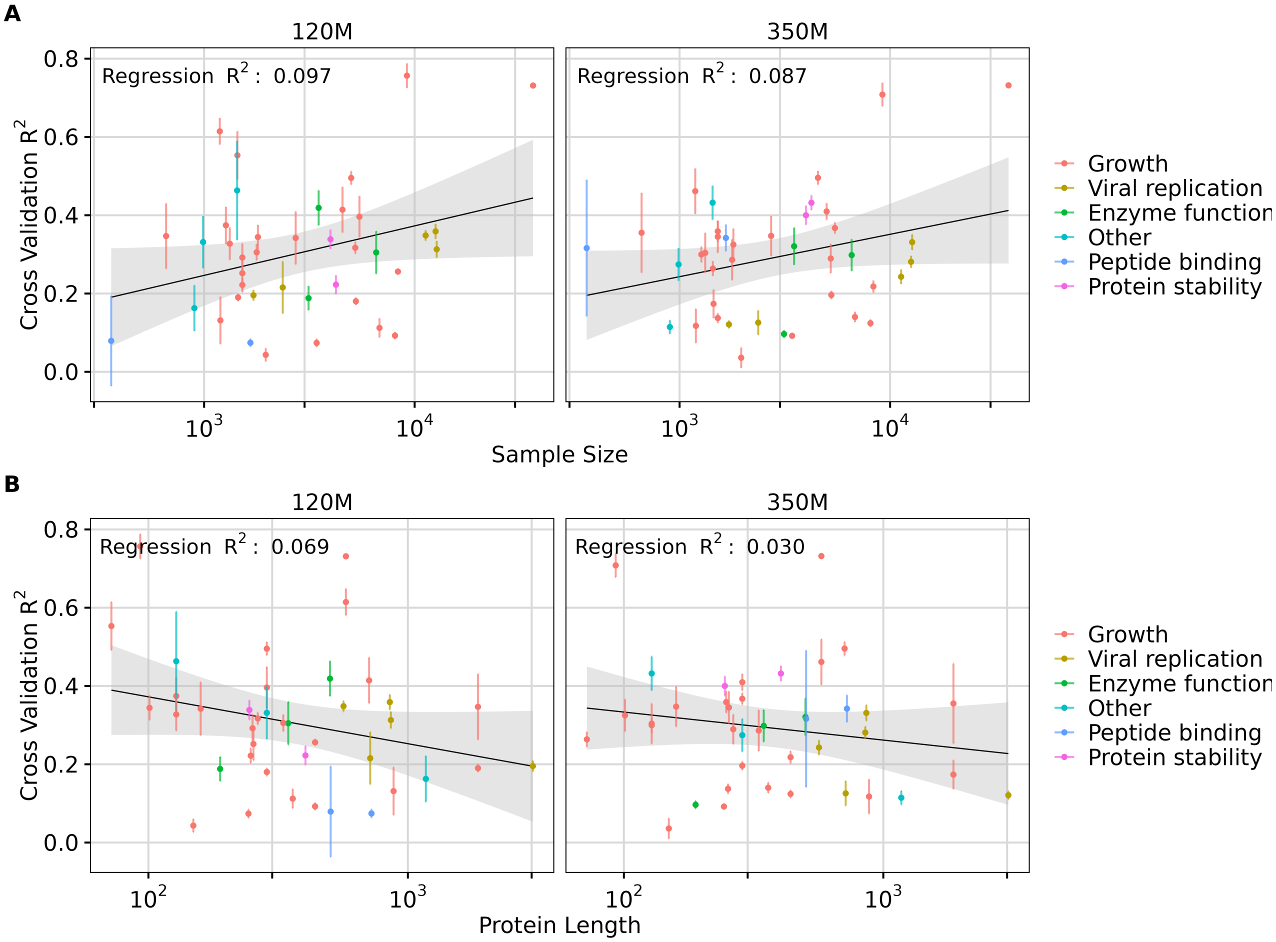

Fig. S8. Effect of sample size and protein length in transfer learning using AMPLIFY embeddings. A) Effect of **sample size** on transfer learning using AMPLIFY models embeddings quantified by the regression results (R^2 score). B) Effect of **protein length** on transfer learning using AMPLIFY models embeddings quantified by the regression results (R^2 score). Each dot represents a dataset, and its color indicates the corresponding data measurement type. In each subplot, the y-axis represents the averaged 5-fold cross-validation R^2 scores, and error bars represent the standard deviation. The left and right panels represent the two model sizes, 120 million and 350 million parameters, respectively.
