# Supplementary material for: Scaling Down for Efficiency: Medium-Sized Transformer Models for Protein Sequence Transfer Learning
Source: bioRxiv. 2024 Nov 24:2024.11.22.624936. Preprint. [Version 1] doi: 10.1101/2024.11.22.624936 (PMC11601519; doi:10.1101/2024.11.22.624936)
Supplement: Supplementary file 1 [file media-1.pdf]

Supporting Information for

Scaling Down for Efficiency: Medium-Sized Transformer Models for Protein Sequence Transfer Learning

Luiz C. Vieira, Morgan L. Handojo and Claus O. Wilke

This PDF file includes:

Figs. S1 to S9

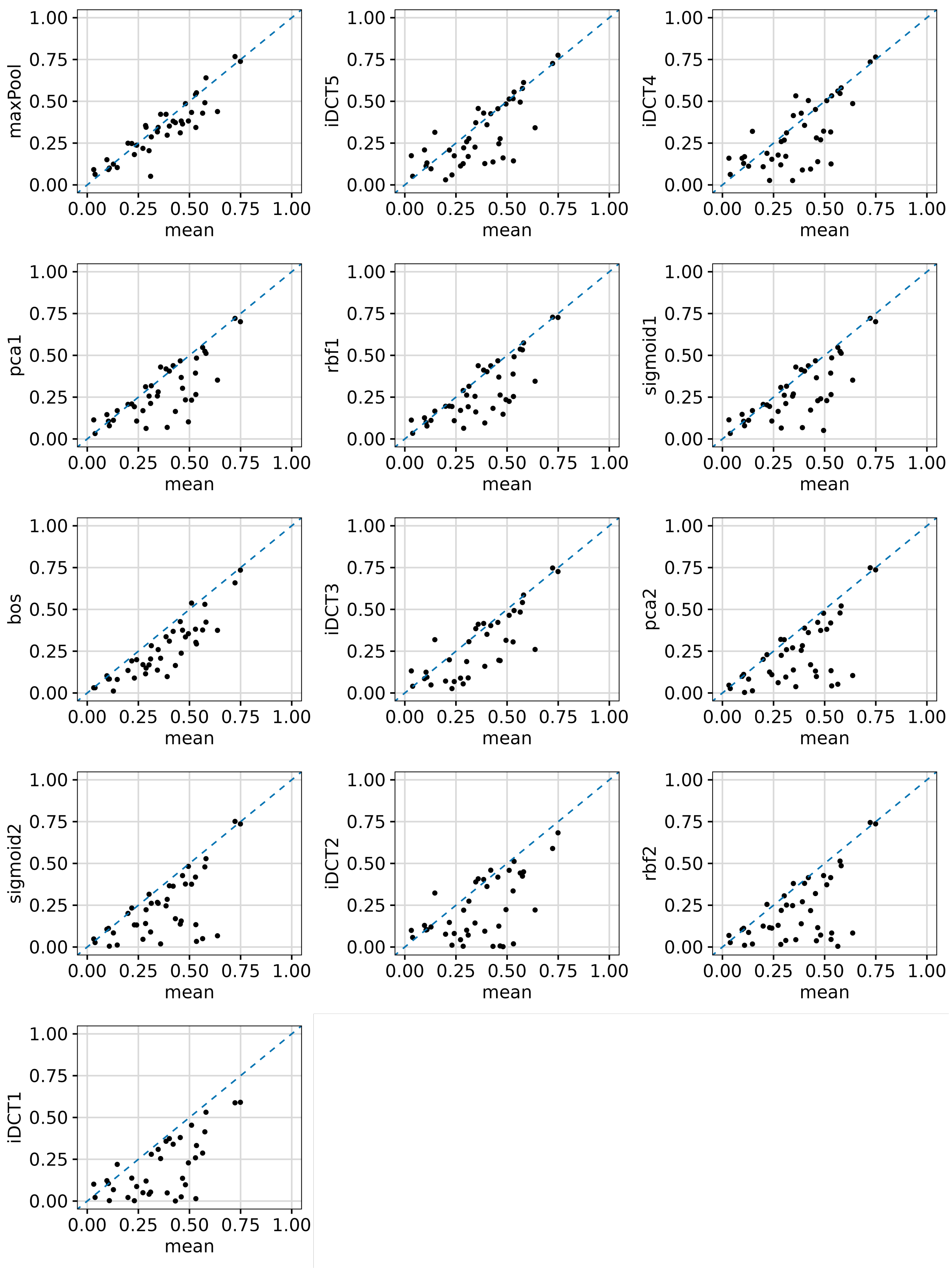

Fig. S1. Comparison of mean embeddings with 13 alternative compression methods on DMS datasets. In each plot, the x-axis represents the averaged R^2 scores from 5 cross-validations for the mean embeddings, while the y-axis shows the averaged R^2 scores from 5 cross-validations for one of the 13 compression methods. Each dot corresponds to one of the 40 DMS datasets analyzed.

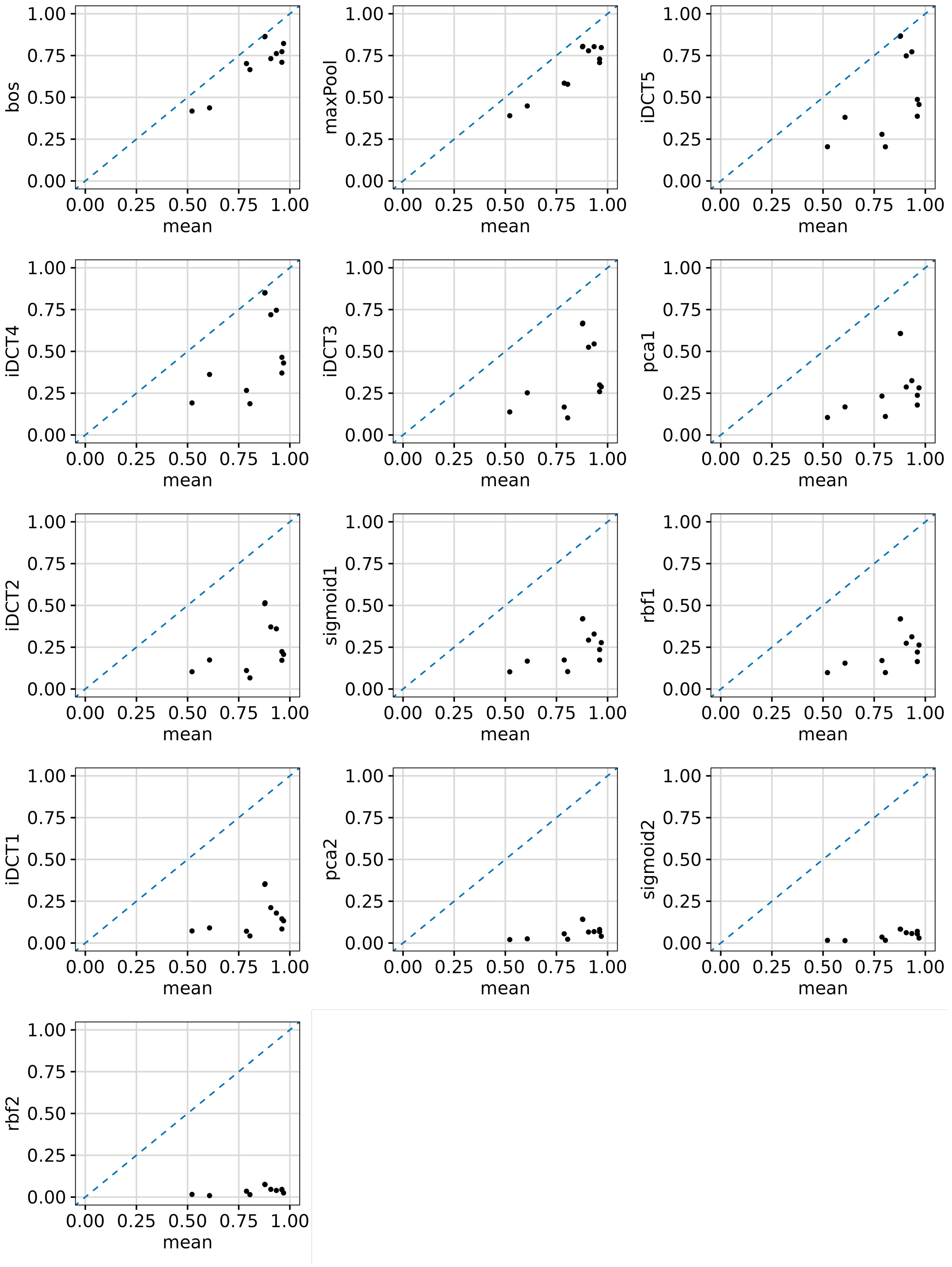

Fig. S2. Comparison of mean embeddings with 13 alternative compression methods on PISCES dataset. In each plot, the x-axis represents the averaged R^2 scores from 5 cross-validations for the mean embeddings, while the y-axis shows the averaged R^2 scores from 5 cross-validations for one of the 13 compression methods. Each dot corresponds to one of the 12 targets analyzed from PISCES dataset (PCPs and Secondary Structure).

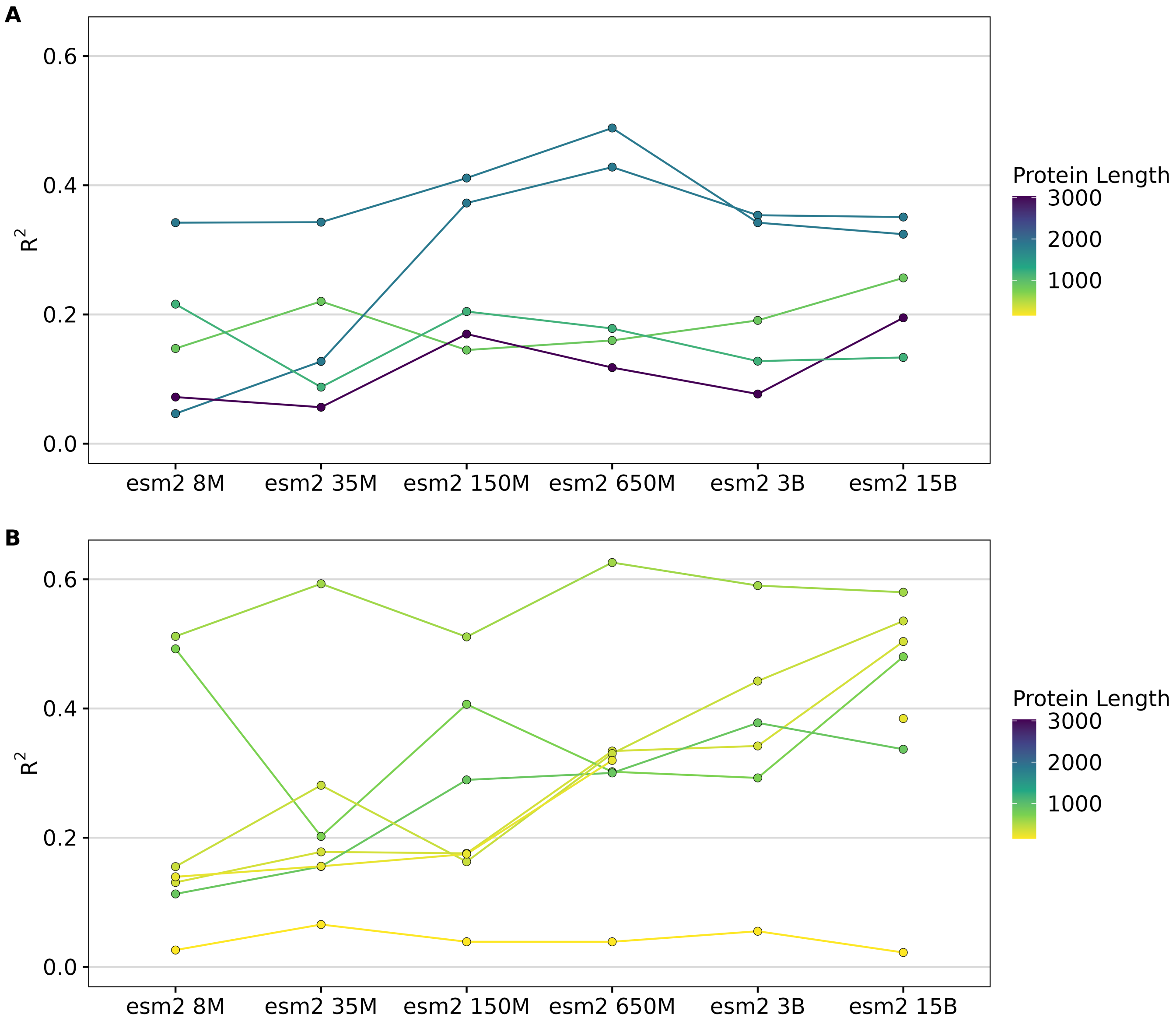

Fig. S3. Performance of ESM2 models on larger protein sequences. A) Prediction scores across 5 different DMS datasets with sequences length greater than 1022. B) Prediction scores across 6 different DMS datasets with sequences length less than 1022. The y-axis represents R^2 scores, and the x-axis shows the different model sizes. Colors represent protein length.

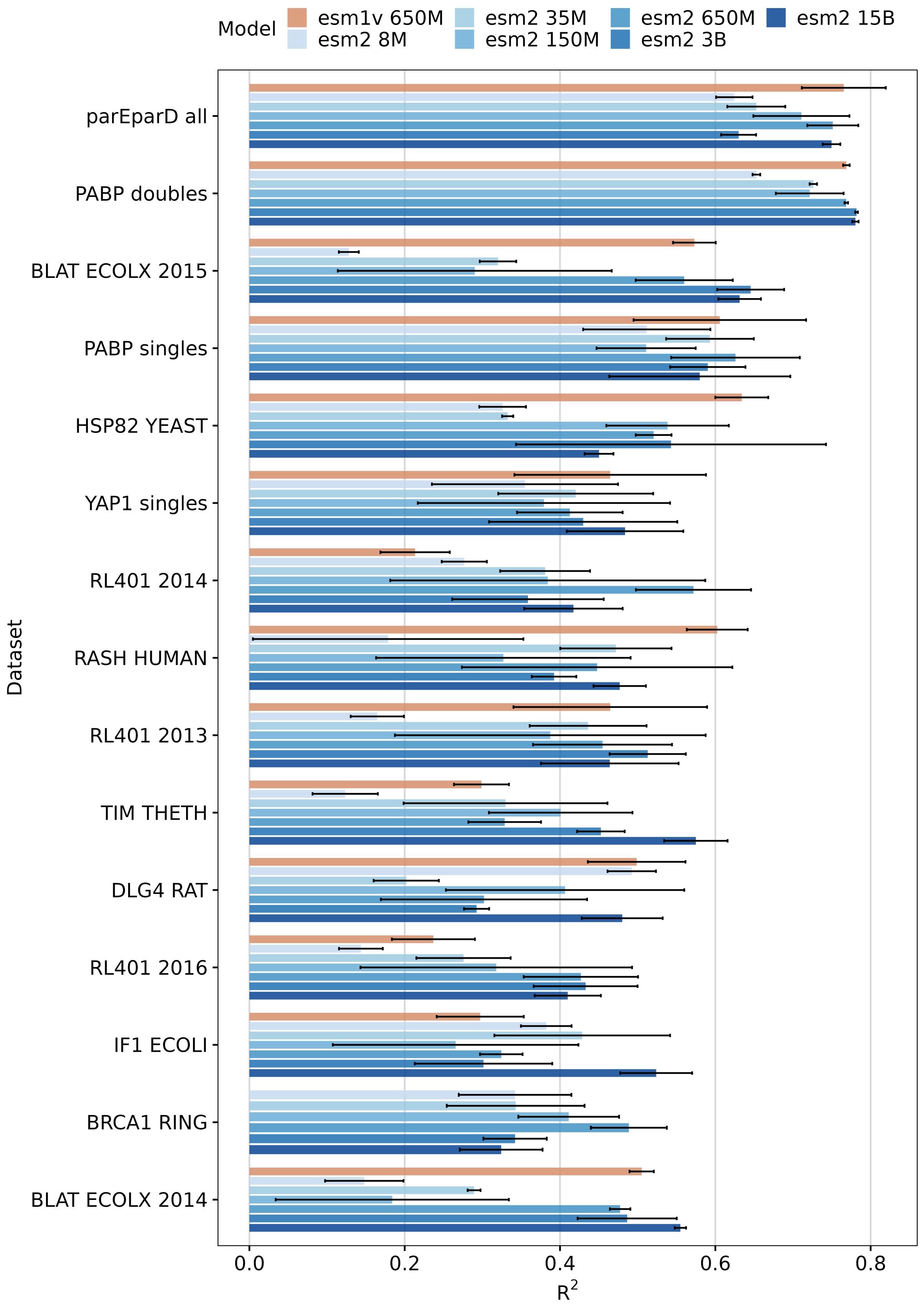

Fig. S4. Impact of ESM Model size on transfer learning by dataset. The x-axis represents the evaluation metric from 5 cross-validation runs, reported as R^2 scores for the test set, while the y-axis displays the top 15 DMS datasets with the highest scores out of the 40 datasets analyzed. Different colors represent the various model sizes.

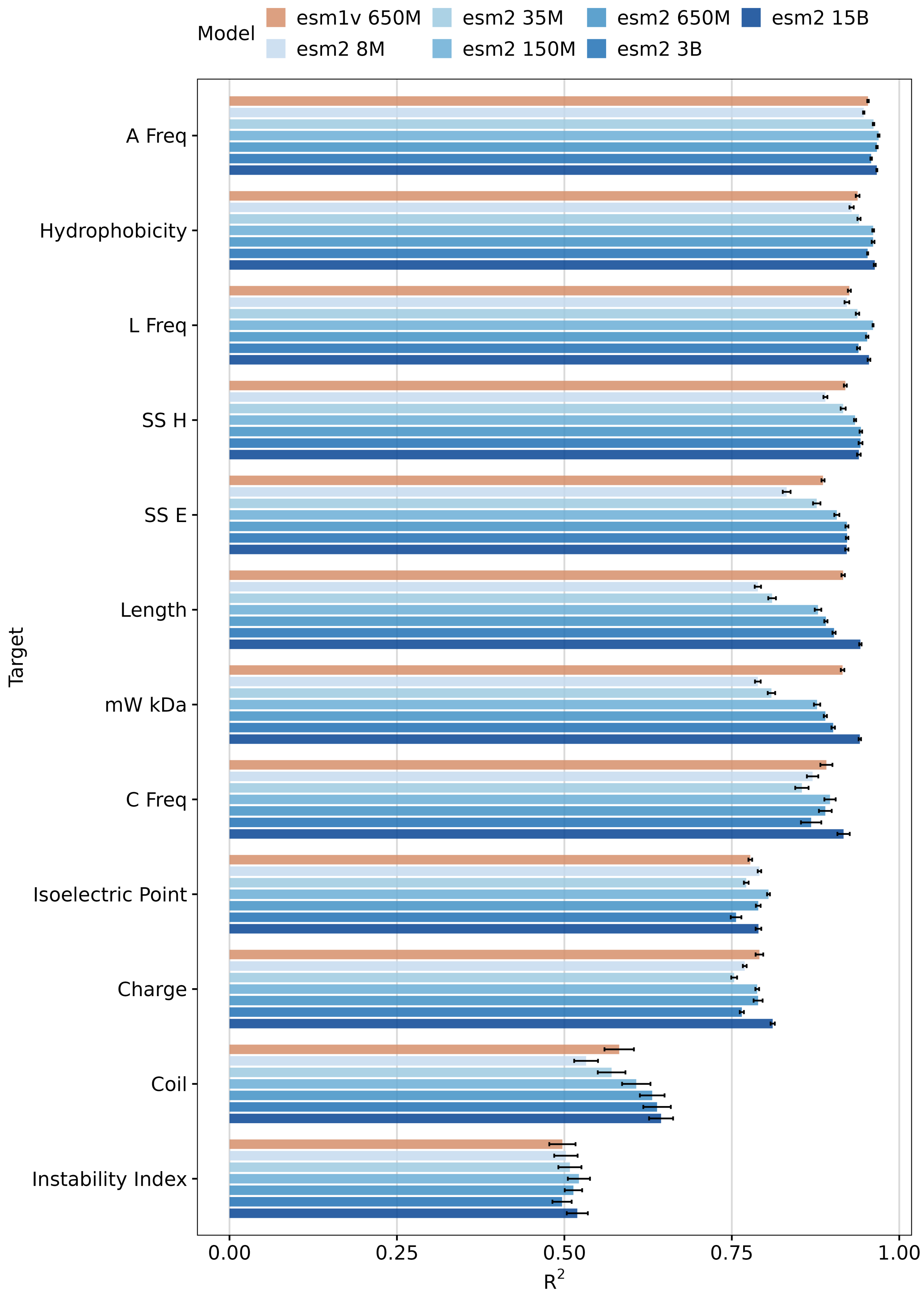

Fig. S5. Impact of ESM Model size on transfer learning by targets. The x-axis represents the evaluation metric from 5 cross-validation runs, reported as R^2 scores for the test set, while the y-axis displays the different targets analyzed from PISCES dataset. Different colors represent different model sizes.

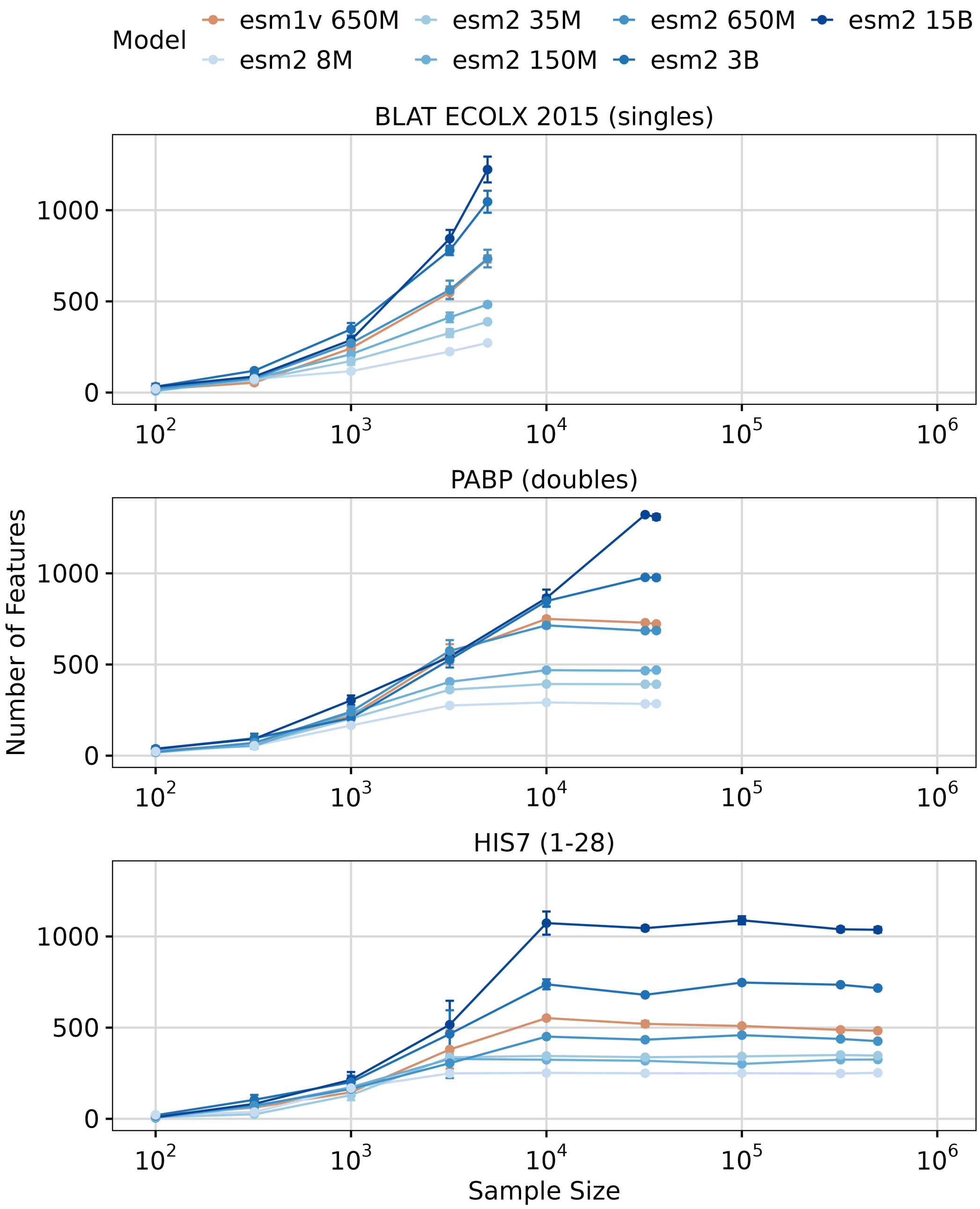

Fig. S6. Number of features boosts performance in protein prediction tasks. Number of important features identified by LassoCV regression for three DMS datasets with varying mutation complexities: single mutations (BLAT ECOLX), double mutations (PABP), and multiple mutations (HIS7 YEAST). The y-axis represents number of important features (or non-zero coefficients set by Lasso l1 penalization), while the x-axis denotes the sample sizes tested. ESM models sizes are represented by colored lines: ESM1v 650M (Orange) and ESM2 8M, 35M, 150M, 650M, 3B, and 15B (Blues)

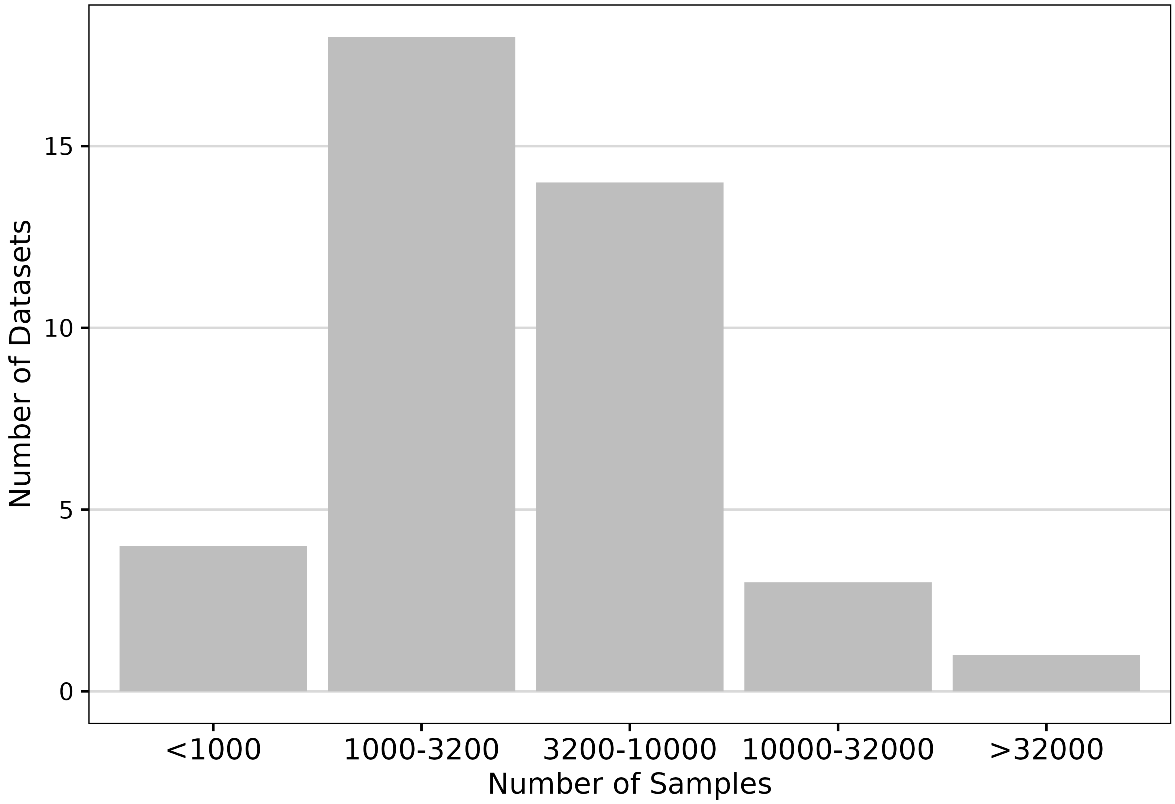

Fig. S7. Sample size distribution. The histogram represents the number of Mutation Scanning datasets datasets grouped by sample size bins.

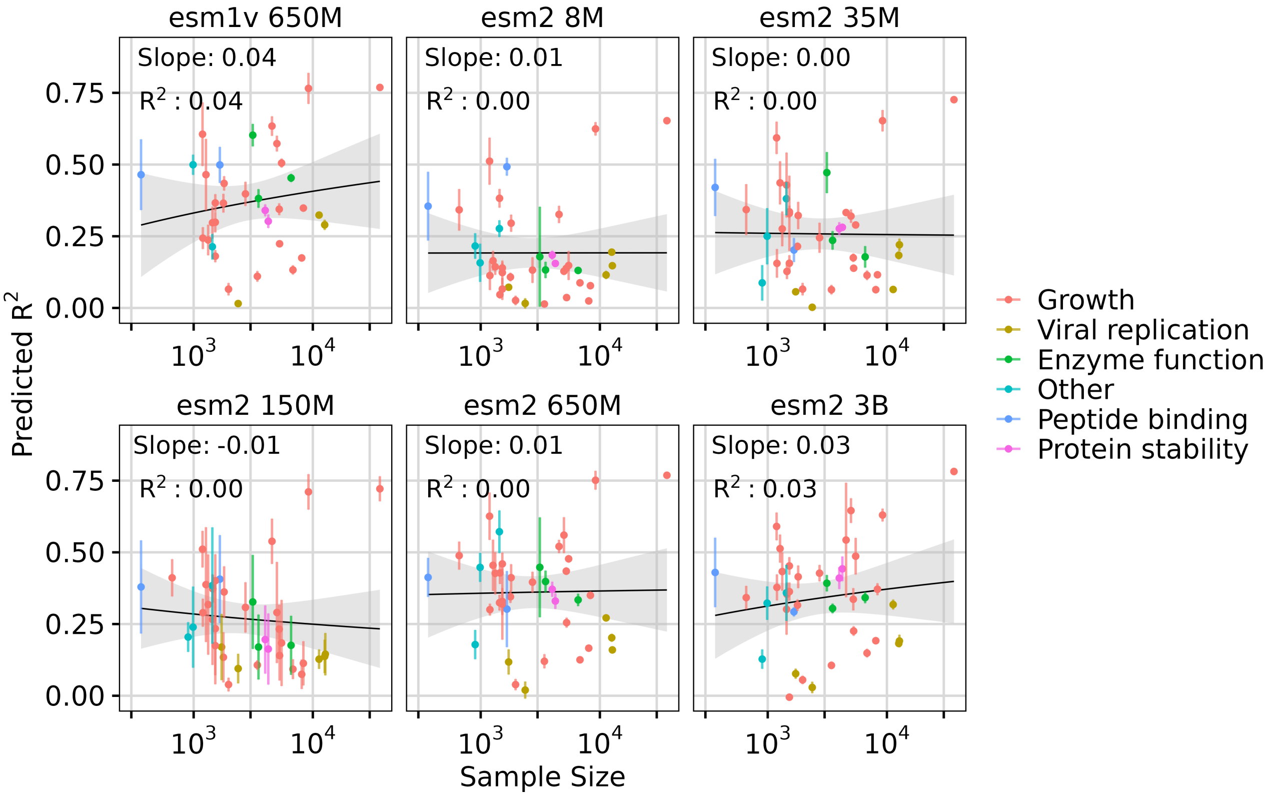

Fig. S8. Effect of sample size on transfer learning. The impact of sample size on transfer learning using ESM model embeddings, quantified by a regression model (slope and R^2 score). The y-axis in each subplot represents the average R^2 score from 5 cross-validation runs on the test set for each of the 40 DMS datasets analyzed. The x-axis in each subplot represents the sample size of each dataset.

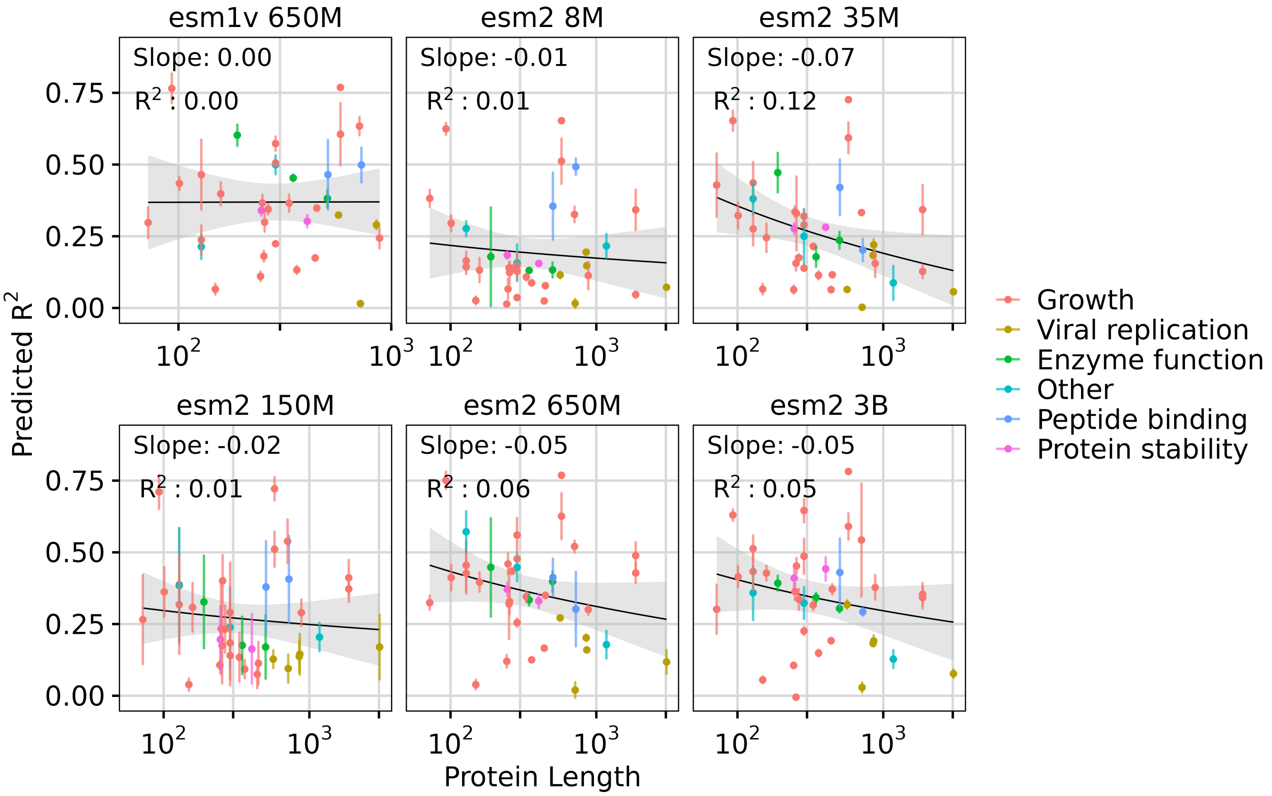

Fig. S9. Effect of protein length on transfer learning. The impact of protein length on transfer learning using ESM model embeddings, quantified by a regression model (slope and R^2 score). The y-axis in each subplot represents the average R^2 score from 5 cross-validation runs on the test set for each of the 40 DMS datasets analyzed. The x-axis in each subplot represents the protein length of each dataset.
